# Intracerebral overexpression of miR-669c is protective in mouse ischemic stroke model by targeting MyD88 and inducing alternative microglial/macrophage activation

**DOI:** 10.1186/s12974-020-01870-w

**Published:** 2020-06-19

**Authors:** Natalia Kolosowska, Maria Gotkiewicz, Hiramani Dhungana, Luca Giudice, Rosalba Giugno, Daphne Box, Mikko T. Huuskonen, Paula Korhonen, Flavia Scoyni, Katja M. Kanninen, Seppo Ylä-Herttuala, Tiia A. Turunen, Mikko P. Turunen, Jari Koistinaho, Tarja Malm

**Affiliations:** 1 University of Eastern Finland, A.I. Virtanen Institute for Molecular Sciences, P.O. Box 1627, FI-70211 Kuopio, Finland; 2 Neuroscience Center, Helsinki Institute of Life Science, University of Helsinki, Helsinki, Finland; 3 Department of Computer Science, University of Verona, Verona, Italy

**Keywords:** Stroke, Neuroinflammation, MicroRNAs, Microglia/macrophage activation, Functional improvement

## Abstract

**Background:**

Ischemic stroke is a devastating disease without a cure. The available treatments for ischemic stroke, thrombolysis by tissue plasminogen activator, and thrombectomy are suitable only to a fraction of patients and thus novel therapeutic approaches are urgently needed. The neuroinflammatory responses elicited secondary to the ischemic attack further aggravate the stroke-induced neuronal damage. It has been demonstrated that these responses are regulated at the level of non-coding RNAs, especially miRNAs.

**Methods:**

We utilized lentiviral vectors to overexpress miR-669c in BV2 microglial cells in order to modulate their polarization. To detect whether the modulation of microglial activation by miR-669c provides protection in a mouse model of transient focal ischemic stroke, miR-669c overexpression was driven by a lentiviral vector injected into the striatum prior to induction of ischemic stroke.

**Results:**

Here, we demonstrate that miR-669c-3p, a member of chromosome 2 miRNA cluster (C2MC), is induced upon hypoxic and excitotoxic conditions in vitro and in two different in vivo models of stroke. Rather than directly regulating the neuronal survival in vitro, miR-669c is capable of attenuating the microglial proinflammatory activation in vitro and inducing the expression of microglial alternative activation markers arginase 1 (Arg1), chitinase-like 3 (Ym1), and peroxisome proliferator-activated receptor gamma (PPAR-γ). Intracerebral overexpression of miR-669c significantly decreased the ischemia-induced cell death and ameliorated the stroke-induced neurological deficits both at 1 and 3 days post injury (dpi). Albeit miR-669c overexpression failed to alter the overall Iba1 protein immunoreactivity, it significantly elevated Arg1 levels in the ischemic brain and increased colocalization of Arg1 and Iba1. Moreover, miR-669c overexpression under cerebral ischemia influenced several morphological characteristics of Iba1 positive cells. We further demonstrate the myeloid differentiation primary response gene 88 (MyD88) transcript as a direct target for miR-669c-3p in vitro and show reduced levels of MyD88 in miR-669c overexpressing ischemic brains in vivo.

**Conclusions:**

Collectively, our data provide the evidence that miR-669c-3p is protective in a mouse model of ischemic stroke through enhancement of the alternative microglial/macrophage activation and inhibition of MyD88 signaling. Our results accentuate the importance of controlling miRNA-regulated responses for the therapeutic benefit in conditions of stroke and neuroinflammation.

## Background

MicroRNAs (miRNAs) are a class of small, non-coding RNA molecules of approximately 22 nucleotides in length that function mainly by post-translational repression or degradation of target mRNAs by binding to their 3′ untranslated regions [1, 2]. The domain at the 5′ end of the miRNA molecule that includes nucleotide position from 2 to 7 is important for mRNA target recognition and has been defined as the miRNA seed. However, miRNAs function is not limited to the classical mechanism of post-translational suppression, as some of these molecules have been shown to target promoter or enhancer regions and thereby control gene expression [3–5].

Several studies have indicated that ischemic stroke alters the expression of multiple miRNAs both in mice and in humans with the capacity to alter cellular stress responses [6–10]. In addition, stroke-induced neuroinflammatory events, especially microglial responses, have been shown to be regulated at the level of miRNAs [11–13]. However, only a handful of studies have demonstrated that these miRNAs can be targeted for therapeutic benefit in the models of cerebral ischemia [12, 14, 15]. Thus, the knowledge on the role of miRNAs in regulation of the stroke-induced neuroinflammatory responses is still limited.

C2MC, also known as miR-297-669 cluster, is derived from intron 10 of the Polycomb group gene sex comb on the midleg with four MBT domains-2 (Sfmbt2) on mouse chromosome 2 [16]. MiRNA members of this cluster have been shown to be upregulated upon different harmful stimuli such as nutrient depletion conditions in Chinese hamster ovary (CHO) cells [17, 18], acetaminophen-induced liver injury [19] and liver aging in mice [20], sodium arsenite exposure in P19 mouse embryonal carcinoma cells [21] and in the ischemic cortex 24 h after transient middle cerebral artery occlusion (tMCAo) in rats [22].

Since some members of miR-297-669 cluster are regulated under various stress conditions including glucose deprivation or cerebral ischemia, the aim of this study was to elucidate how a member of C2MC, miR-669c, is implicated in the pathology of ischemic stroke, and to evaluate the potential role of this miRNA in the regulation of stroke-induced neuroinflammatory events. Here, we show that miR-669c-3p is induced upon brain ischemia, its intracerebral overexpression modulates microglial/macrophage activation and it directly targets the myeloid differentiation primary response gene 88 (MyD88), the canonical adaptor protein implicated in toll-like receptor (TLR) and IL-1 signaling, critical in mediating innate immune responses. Lentiviral vector-mediated overexpression of miR-669c was protective in a mouse model of tMCAo through miR-669c-mediated suppression of proinflammatory responses and concomitant enhancement of microglial/macrophage alternative activation. Based on our data, we propose that miR-669c-3p overexpression represents a novel therapeutic approach for the treatment of cerebral ischemia.

## Methods

### Primary cortical neuron cultures and glutamate excitotoxicity assay

Primary cortical neuron cultures were prepared from C57BL/6 J embryonic day 15 embryos as described in Malm et al. [23]. Briefly, cortices were dissected and the tissue was dissociated with trypsin for 15 min at 37 °C (0.0125%, Sigma-Aldrich, St. Louis, USA). Isolated neurons were seeded at a density of 125 × 10^3^ cells/well on 48-well or 1.8 × 10^6^ cells/well on 6-well plate format pre-coated with poly-D-lysine (Sigma-Aldrich, St. Louis, USA) in complete neurobasal media containing 2% B27 supplement, 500 μM l-glutamine and 10 μg/ml gentamycin (all reagents ThermoFisher Scientific, Waltham, USA). On 5th day in vitro (DIV) after seeding 50% of the media was changed and the cultures were used for experiments on 6 DIV*.* Cells were treated with 400 μM glutamate (Sigma-Aldrich, St. Louis, USA) for 24 h prior to the measurements of cell viability by the MTT assay or RNA isolation.

### Primary microglia and astrocyte cultures

Primary microglial cultures were prepared from C57BL/6 J neonatal mice of 0–3 postnatal days as described elsewhere [23]. Briefly, the mice were sacrificed by decapitation and the brains were dissected. The tissue was mechanically dissociated and incubated in DMEM/F-12 supplemented with 1% penicillin/streptomycin and 0.05% trypsin-EDTA (all ThermoFisher Scientific, Waltham, USA). Trypsin activity was inactivated with complete media DMEM/F-12 containing 10% heat-inactivated fetal bovine serum (iFBS) and 1% penicillin/streptomycin (all ThermoFisher Scientific, Waltham, USA), the tissue was homogenized and plated on 15 cm diameter cell culture dishes and left at culture at 37 °C, 5% CO_2_ for 3 weeks. Thereafter, the astrocyte layer from mixed glial culture was trypsinized, collected, and seeded on poly-l-lysine (Sigma-Aldrich, St. Louis, USA) pre-coated T75 flasks. Remaining microglia were collected and directly plated on 48-well or 6-well plate format at the density of 125 × 10^3^ cells/well and 1 × 10^6^ cells/well, respectively.

### N2a cell cultures and oxygen-glucose deprivation/reoxygenation (OGD/R)

Mouse Neuro2a (N2a) cell line was seeded at a density of 37.5 × 10^3^ cells/well on 48-well or 3 × 10^5^ cells/well on 6-well plate format in complete DMEM with GlutaMAX-1 containing d-glucose (25 mM) and sodium pyruvate (1 mM) supplemented with 10% iFBS and 1% penicillin-streptomycin (all reagents ThermoFisher Scientific, Waltham, USA). For the OGD/R exposures, 24 h after plating the cells, media were replaced with DMEM depleted from glucose and sodium pyruvate but otherwise supplemented as the standard culture media (ThermoFisher Scientific, Waltham, USA). For induction of hypoxia, the cells were incubated in the hypoxia chamber in 1% O_2_ and 5% CO_2_ (ProOx C21, Biospherix Ltd., Parish, USA) for 1, 2, or 3 h, after which the media was changed to complete DMEM and cells returned into the normal incubator for 24 h reoxygenation. The control plates were treated similarly, but instead maintained in high glucose DMEM media in a regular CO_2_ incubator. Passages 2-10 were used for the experiments. Cell line was negatively tested for mycoplasma with MycoAlert Mycoplasma Detection Kit (Lonza, Basel, Switzerland).

### BV2 microglia

Mouse microglial BV2 cells were seeded at a density of 3 × 10^5^ cells/well on 6-well plate format in complete RPMI-1640 media (Sigma-Aldrich, St. Louis, USA) supplemented with 1% GlutaMAX, 10% iFBS, and 5 μg/ml gentamycin (all reagents ThermoFisher Scientific, Waltham, USA). The cells were used for the experiments 24 h post seeding. BV2 cells were treated with lipopolysaccharide (LPS #L2630, serotype O111:B4, Sigma-Aldrich, St. Louis, USA), 20 ng/ml or mouse recombinant interleukin 4 (IL-4, PeproTech, Rocky Hill, USA), or 20 ng/ml IFN-γ in complete media for 24 h. Passages 2-10 were used for the experiments. Cell line was negatively tested for mycoplasma with MycoAlert Mycoplasma Detection Kit (Lonza, Basel, Switzerland).

### Lentiviral constructs

Pre-miR-669c hairpin together with its downstream and upstream flanking genomic sequence of 258 bp in length was cloned into third-generation human immunodeficiency virus 1 (HIV-1)-based LV-PGK-GFP-U6-miRNA vector. Control vector contained only a green fluorescent protein (GFP) sequence. Vectors were prepared by standard calcium phosphate transfection method in 293 T cells as described previously [24]. Lentiviral constructs used in this study were produced by the BioCenter Kuopio National Virus Vector Laboratory in Kuopio, Finland.

### Lentiviral vector transduction in cell cultures

N2a or BV2 cells were seeded at a density of 12 × 10^4^ cells/well on 12-well plate format and the lentiviral vector transduction was performed 24 h after seeding the cells. Lentiviral vectors LV1-GFP (control) or LV1-miR-669c were added into the fresh cell culture media to obtain a multiplicity of infection (MOI) of 30. Transduction was performed for 24 h after which the media containing the lentiviral particles were removed, and the cells visualized under a fluorescent microscope (Carl Zeiss AG, Jena, Germany) to confirm all (99-100%) the transduced cells from both groups were expressing GFP. In addition, GFP expressing cells from both of the transduced groups were sorted with BD FACSAria III to establish the cell lines stably expressing GPF (control) or GFP and miR-669c.

### Permanent middle cerebral artery occlusion (pMCAo) and transient middle cerebral artery occlusion (tMCAo) in mice

To evaluate the levels of miR-669c expression in ischemic conditions in vivo, we utilized two different mouse models of ischemic stroke, the pMCAo and tMCAo. For pMCAo, a total of 15 three-to-six-month-old Balb/cOlaHsd male mice (Harlan Laboratories B.V., An Venrey, Netherlands) were subjected to pMCAo as described before [25]. Briefly, for anesthesia induction, the mice were anesthetized with 5% isoflurane in 30% O_2_/70% N_2_O and during the surgery isoflurane was maintained at 2%. Temperature of the animals was maintained at 37 ± 0.5 °C by a thermostatically controlled system connected to a heating blanket and a rectal probe (Harvard apparatus; PanLab, Cornella, Spain). Incision was made between the ear and the eye to expose the temporal muscle, which thereafter was moved aside. Approximately, a 1-mm hole was drilled on the bone under the muscle to expose the MCA. The dura was removed, the artery gently lifted using forceps, and occluded with a thermocoagulator (Aaron Medical Industries Inc., Clearwater, USA). MCA occlusion was confirmed by cutting the artery, then the temporal muscle was repositioned, and the skin was sutured. The mice were moved to their home cages to recover from the surgery. The animals were sacrificed either 1 or 3 days post ischemia (dpi) for the evaluation of miR-669-3p expression (*N* = 7 per group for 1 dpi and *N* = 8 per group for 3 dpi). To induce tMCAo, the intraluminal middle cerebral artery occlusion model was used as described previously [26]. The animals were initially anesthetized with 5% isoflurane in 30% O_2_/70% N_2_O, while the surgical anesthesia was maintained at 2% isoflurane. Temperature of the animals was maintained at 37 ± 0.5 °C by a homeothermic control system connected to a heating blanket and a rectal probe (Harvard apparatus; PanLab, Cornella, Spain). For tMCAo surgery, a midline neck incision was made and the left common carotid artery (CCA) was ligated proximally to the bifurcation of the internal carotid artery (ICA) and external carotid artery (ECA). Then the left ECA was isolated, ligated, and a suture was made around the ICA. Subsequently, a small cut was made in the CCA and a 20 mm silicone intraluminal monofilament with a diameter of 0.21 ± 0.02 mm (#602156PK10Re, Doccol Corporation, USA) was introduced through the incision and inserted further until a slight resistance was felt, confirming the middle cerebral artery (MCA) occlusion. An additional suture around the ICA was made to fix the filament in the correct position. After 45 min of the occlusion time, during which the anesthesia was maintained at 1% isoflurane, the filament was withdrawn, and the ICA was ligated. In the sham-operated animals, the occluding filament was inserted only 5 mm above the carotid bifurcation. Analgesic buprenorphine (Temgesic, Schering-Plough, Belgium) was administered once in the concentration of 0.03 mg/kg IP immediately after the surgery. The mice were transferred to a heated recovery box for 2 h. After that, animals received water-softened food pellets to facilitate their feeding. A total number of 54 four-month-old C57BL/6 J male mice were used. The animals were sacrificed either 1 or 3 days post ischemia (dpi) for the evaluation of the expression of miR-669-3p (*N* = 3 per group for 1 dpi and *N* = 3 per group for 3 dpi).

### Intracerebral lentiviral vector injections

To evaluate whether lentivirally driven miR-669c overexpression provides protection against tMCAo in mice, C57BL/6 J males were intrastriatally injected with lentiviral vector encoding for miR-669c or control GFP vector. The tMCAo model of ischemic stroke was chosen since it produces cortico-striatal lesion, whereas pMCAo leads to strictly cortical lesion and lentiviral vector injections were easier to perform into the striatum rather than into the cortex. Briefly, three-month-old C57BL/6 J male mice were randomized into four treatment groups using GraphPad QuickCalcs (www.graphpad.com/quickcalcs/, GraphPad Software, San Diego, CA, USA): sham- or tMCAo-operated animals injected with either LV1-GFP or LV1-miR-669c. Animals were initially anesthetized by 5% isoflurane in 30% O_2_/70% N_2_O and placed on a heating pad (Harvard apparatus, PanLab, Cornella, Spain) connected with a rectal probe to maintain the body temperature at 37 ± 0.5 °C. The surgical anesthesia was maintained using 2% isoflurane and the mouse head was fixed in a stereotaxic apparatus (Kopf Instruments, Tujunga, USA). Thereafter, a burr hole was drilled 1.8 mm left lateral to the sagittal suture and 0.4 mm posterior to the bregma. A blunt needle of a 10 μl Hamilton syringe was inserted 2.9 mm deep into the striatum (caudate putamen) under the cortex. Depending on the group, 1 μl of LV1-GFP or LV1-miR-669c, both containing 2.28 × 10^9^ transducing units (TU) per ml, was injected into the caudate putamen at a rate of 0.2 μl/min using a micro-infusion pump (Harvard Apparatus, Holliston, USA). To allow the pressure equilibration and to prevent backflow of the injected LV suspension, the needle was retracted 10 min post injection, then the hole was sealed with bone wax, and the scalp wound was closed with Ethilon nylon sutures (Ethicon Inc., USA). For the post-surgery analgesia buprenorphine solution was injected intraperitoneally (IP) at 0.03 mg/kg (Temgesic, Schering-Plough, Belgium). Three weeks after the lentiviral vector injections the mice were subjected to tMCAo as described above. Surgeries were performed as blinded to the study groups. A total number of 54 three-month-old C57BL/6 J male mice were used (*N* = 11 in sham-operated animals, *N* = 18 for LV1-GFP, and *N* = 18 LV1-miR-669c stroke groups). The mice were sacrificed at 3 dpi for further analyses.

### Behavioral testing with composite neuroscore

At 1 and 3 dpi mice which underwent tMCAo were scored for the neurological function according to general and focal neurological scale as previously described by Clark et al. [27]. Briefly, for the general assessment, the condition of fur, ears, eyes, posture, spontaneous activity (scored between 0 and 4), and possible epileptic behavior (score 0-12) were determined. In this scoring, 0 meant the animal was displaying normal, healthy behavior and 4 or 12 indicated very severe neurological deficits. In the scoring for focal deficits the body symmetry, gait, climbing ability on the 45° angle grip surface, circling behavior, front limb symmetry, compulsory circling, as well as whisker and ear response were assessed on a 0-4 scale. In this test score, 0 corresponded to no deficits and 4 indicated severe impairment. Behavioral tests were performed as blinded to the study groups (*N* = 11 in sham-operated animals, *N* = 18 for LV1-GFP, and *N* = 18 LV1-miR-669c stroke groups).

### Magnetic resonance imaging (MRI)

MRI was performed at 1 and 3 dpi in the mice anesthetized with 1.8% isoflurane in 30% O_2_/70% N_2_O, to determine the lesion volume using a horizontal 9.4 T Oxford NMR 400 magnet (Oxford instrument PLC, Abington, UK) interfaced with Agilent Direct Drive console as previously described [25]. Multi-slice T2-weighted images were acquired with echo time/repetition time of 40 ms/3000 ms, matrix size 128 × 256, field of view 19.2 × 19.2 mm^2^, slice thickness 0.8 mm, and number of slices 12. Images were analyzed using the Aedes software (Kuopio, Finland) for MatLab program (Math-works, Natick, USA). The following formula was used to calculate the lesion volume: Lesion volume = (volume of contralateral hemisphere–(volume of ipsilateral hemisphere–volume of the lesion))/volume of contralateral hemisphere, as previously described [28]. The lesion volume is expressed as percentage. Analyses were performed as blinded to the study groups (*N* = 10 for LV1-GFP and *N* = 11 LV1-miR-669c stroke groups).

### Immunohistochemistry

Anesthetized mice were perfused transcardially with cold heparinized (2500 IU/l; Heparin LEO 5000 IU/ml, Leo Pharma A/S, Ballerup, Denmark) saline, their brains were dissected and fixed in 4% paraformaldehyde solution in 0.1 M phosphate buffer (PB) pH 7.4. After 18-20 h of postfixation, the brains were cryoprotected in 30% sucrose in PB for 48 h and frozen in liquid nitrogen. Thereafter, the brains were stored in −70 °C until cryosectioning. Six 20 μm coronal brain sections each 400 μm apart were cut using a cryostat (Leica Microsystems, Wetzlar, Germany), collected on superfrost microscope slides (ThermoFisher Scientific, Waltham, USA) and stored at −70 °C until analysis. After washing with phosphate-buffered saline (PBS) pH 7.4 and PBST containing 0.05% Tween-20 (Sigma-Aldrich, St. Louis, USA), sections were blocked by 1 h incubation in 10% normal goat or rabbit serum (NGS or NRS; Vector Laboratories Ltd., Burlingame, USA). The following primary antibodies were incubated overnight at room temperature (RT): rabbit anti-Iba1 (ionized calcium-binding adapter molecule 1, dilution 1:250; Wako PureChemical Industries Ltd., Tokyo, Japan), goat anti-Arg1 (dilution 1:300; Santa Cruz Biotechnology, Dallas, USA), rat anti-CD45 (leukocyte common antigen, dilution 1:200; Bio-Rad, Hercules, USA), and rat anti-MyD88 (dilution 1:100; R&D Systems, Minneapolis, USA) in 5% NGS or NRS. For double IHC stainings, antibodies Iba1 and Arg1, and Arg1 together with CD45 were used. For antigen retrieval prior to incubation with primary antibodies, the sections were incubated for 1 h in preheated (92 °C) 10 mM citrate buffer, pH 6.0. After washing in PBST, the sections were incubated with Alexa Fluor 488, 568, or 647 secondary antibody (dilution 1:500; ThermoFisher Scientific, Waltham, USA) for 2 h at RT, washed again, air dried, and mounted in Vectashield with DAPI (Vector Laboratories Ltd., Burlingame, USA). Negative controls were included in parallel sessions, following the same procedures, except for the incubation with primary antibodies. For the analyses, entire sections were imaged with × 5 magnification on Zeiss Axio Imager 2 coupled to Axiocam digital camera and using the Zen software (all Carl Zeiss AG, Jena, Germany). The confocal images were acquired from ipsilateral striatum (caudate putamen) under × 20 or × 40 magnification with Zeiss Axio Observer with Zeiss LSM 800 Airyscan confocal module (Carl Zeiss AG, Jena, Germany). Immunoreactivities were quantified using the ImageJ software (National Institute of Health, USA) and measured as the relative immunoreactive area for Iba1, Arg1, CD45, or MyD88. For analysis of the proportion of Arg1^+^ to round in shape, CD45^+^ cells, the × 20 confocal images were taken from three slices per animal, from the ipsilateral striatum area with the highest observed Arg1 immunoreactivity. Colocalization of Iba1 and Arg1 was quantified from × 20 confocal images using the ImageJ software (National Institute of Health, USA) with JACoP plugin, as described elsewhere [29]. Pearson’s correlation coefficient, representing relationship between Iba1 and Arg1 channel intensity distribution, and Mander’s overlap coefficient M2, describing the fraction of Arg1^+^ cells that colocalize with Iba1^+^ cells, were calculated from four slices per animal using threshold values of 50 for Arg1 and 100 for Iba1 immunoreactivities, respectively. Analyses were performed as blinded to the study groups (*N* = 3 in sham or *N* = 5-6 in each stroke group).

### Fluorescent in situ hybridization

The localization of miR-669c-3p in the brains of LV1-GFP and LV1-miR-669c injected mice was evaluated by FISH using ViewRNA miRNA ISH Cell Assay Kit (ThermoFisher Scientific, Waltham, USA) according to manufacturer’s protocol with modifications. Briefly, the sections were washed with PBS, incubated in preheated 10 mM citrate buffer pH 6.0 for antigen retrieval, cross-linked with EDC solution and permeabilized with Detergent Solution QC, followed by target hybridization, signal amplification, and detection. Negative controls were included in parallel sessions following the same procedures, except for the incubation with miR-669c-3p specific probe. Thereafter, sections were incubated with primary antibody anti-Iba1 and then secondary antibody Alexa Fluor 647 as described above. Entire sections were imaged with × 10 magnification on Zeiss Axio Imager 2 coupled to Axiocam digital camera and using the Zen software (all Carl Zeiss AG, Jena, Germany).

### Microglial/macrophage (Iba1 positive) cell morphology analyses

The morphological analysis of Iba1 expressing cells was done at 3 dpi as previously described [30] with some modifications. Briefly, the cell area, perimeter, area/perimeter ratio, compactness, solidity, eccentricity, EquivDiameter, circularity, and roundness were measured using the ImageJ software (National Institute of Health, USA) with Analyze particles command. The images for analysis were captured under × 40 magnification with Zeiss Axio Imager 2 coupled to Axiocam digital camera and using the Zen software (all Carl Zeiss AG, Jena, Germany).

### Cytokine secretion measurements

Prior to transcardial perfusion, a 300 μL blood sample for isolation of plasma was withdrawn directly from the heart right ventricle. Buffered 129 mM sodium citrate was used as an anticoagulant in the volume ratio 1:9 of anticoagulant to blood. Collected blood samples were immediately centrifuged at 1500 g for 15 min, and plasma supernatants were additionally spun down at 13,000 g for 2 min to remove any trace of platelets. Plasma samples were aliquoted and stored at −70 °C until analysis. Following the dissection of the brains, the contra- and ipsilateral hemispheres were snap frozen in liquid nitrogen and then homogenized in cold lysis buffer containing 20 mM Tris pH 7.5, 250 mM sucrose, 5 mM EDTA and 10 mM EGTA, prepared in nuclease-free water with complete protease and phosphatase inhibitors cocktail (Sigma-Aldrich, St. Louis, USA). Half of the homogenate was processed for protein isolation and the other half for RNA isolation. The cytometric bead array (CBA) mouse inflammation kit (BD Biosciences, Franklin Lakes, NJ) was used to analyze the levels of IL-6, IL-10, MCP-1, IFN-γ, TNF-α, and IL-12p70 in mouse brain homogenates, plasma samples, and cell culture supernatants according to manufacturer’s instructions. Data was acquired using CytoFLEX S (Beckman Coulter, Indianapolis, USA) and analyzed by FCAP Array software (Soft Flow Hungary Ltd, Pécs, Hungary). Total protein concentrations were determined by BCA Protein Assay Kit (Pierce, Rockford, USA), and the results were used to normalize the CBA data.

### Quantitative real-time PCR (qPCR) analysis of mRNA levels

Total RNA was isolated from cell cultures using the mirVana miRNA Isolation Kit (ThermoFisher Scientific, Waltham, USA). The concentration and purity of RNA samples were determined using NanoDrop 2000 (Thermo Fisher Scientific). Reverse transcription was performed with 500 ng of total RNA, maxima reverse transcriptase, random hexamer primers, and dNTPs in the presence of ribonuclease inhibitor (all reagents ThermoFisher Scientific, Waltham, USA). The final cDNA concentration used for the gene expression analyses was 2.5 ng/μL. The relative expression levels of mRNAs encoding the selected genes were analyzed in duplicates and measured according to the manufacturer protocols by qPCR (StepOnePlus Real-Time PCR System, ThermoFisher Scientific, Waltham, USA) using the following specific TaqMan gene expression assays (ThermoFisher Scientific, Waltham, USA): Aif1 (Mm00479862_g1), Cx3cr1 (Mm00438354_m1), Mmp9 (Mm00442991_m1), Tnfa (Mm00443258_m1), Il6 (Mm00446190_m1), Il1b (Mm00434228_m1), Ccl2 (Mm004412422_m1), Arg1 (Mm00475988_m1), Chil3 (Mm00657889_mH), Pparg (Mm01184322_m1), Il10 (Mm00439614_m1), Tgfb1 (Mm01178820_m1), Myd88 (Mm00440338_m1), Tlr4 (Mm00445273_m1), and Irak4 (Mm00459443_m1). Results were normalized to the levels of endogenous controls: eukaryotic 18S rRNA (TaqMan Ribosomal RNA Control Reagents, #4308329) or GAPDH (#4352932E). Relative mRNA expression was calculated with the 2^−∆∆Ct^ method where Ct is the threshold cycle number and results presented as values in relation to the control conditions.

### Quantitative real-time PCR analysis of miRNA levels

Similar to the quantification of mRNA expression, the total RNA isolated from cell cultures or tissue homogenates using the mirVana miRNA Isolation Kit (ThermoFisher Scientific, Waltham, USA) was used for miRNA expression analyses. Reverse transcription was done with 10 ng of total RNA with TaqMan MicroRNA Reverse Transcription Kit (ThermoFisher Scientific, Waltham, USA), according to the manufacturer’s protocol. QPCR was performed with TaqMan MicroRNA assays (StepOnePlus Real-Time PCR System, ThermoFisher Scientific, Waltham, USA), and the absolute copy number was quantified from the standard curve equation. Standard curve was prepared with a serial dilution of the synthetic ssRNA in 0,1X TE buffer with the siRNA dilution ranging from 0.5 pM to 5 nM and representing from 400 to 4 × 10^6^ copies, respectively.

### Network analysis for miR-669c-3p predicted targets and pathway enrichment analysis

To decipher the most prominent targets for miR-669c-3p, a network analysis was carried out for the predicted targets of miR-669c-3p. MiR-669c-3p targets were retrieved using miRTarBase [31] and TargetScan. MiRTarBase reports miRNA-target interactions validated experimentally by reporter assay, Western blot, microarray, and next-generation sequencing experiments. TargetScan predicts biological targets of miRNAs by searching for the presence of conserved 8mer, 7mer, and 6mer sites that match the seed region of a miRNA [32, 33]. Then, it provides a context score for the confidence of prediction which is the sum of the contribution of multiple features, calculated as in Agarwal et al., 2015 [34]. Precisely, it includes a series of features such as the site type, local AU, distance, sRNA1A, ORF 8mer count, and UTR offset 6mer count. The most confident 15% of the TargetScan genes associated with miR-669c-3p were included into the analysis. Then the STRING database [35] was utilized to create a network with high confidence (score ≥ 900) STRING interactions between the miRTarBase and TargetScan targets. The network was composed by 29 connected components. The pathway enrichment analysis was performed using the R package ReactomePA [36] for each component. Only the pathways enriched with an adjusted *p* value using the Bonferroni correction lower than 0.01 were retrieved. Six components were shown to be neuroinflammation-associated, according to the involvement of one of these six TargetScan genes: Mdga1, Fbxw11, Igfbp4, Foxo1, Cxcr1, and MyD88. In order to prioritize these genes, the network was expanded with the relationships among both the TargetScan and miRTarBase targets. Finally, the pathway enrichment analysis was repeated.

### RNA pulldown with biotinylated miRNA mimics (miRNA pulldown)

Cell cultures were seeded at a density of 3 × 10^6^ N2a cells/dish or 2 × 10^6^ BV2 cells/dish on 10 cm diameter dishes in complete DMEM or complete RPMI-1640 media, respectively. RNA pulldown was performed as previously described [37] with minor modifications. Briefly, biotinylated mmu-miR-669c-3p and biotinylated control cel-miR-39-3p (both miRCURY LNA microRNA mimics, Premium, Biotin, Exiqon) in 50 nM concentration were used for the transfection. Transfections were performed using Viromer Blue (Lipocalyx GmbH, Halle, Germany) in Opti-MEM media (ThermoFisher Scientific, Waltham, USA) for 4 h after which the transfection media were changed for complete DMEM or complete RPMI-1640 for 20 h. The transfected N2a cells were exposed to 2 h of OGD followed by 24 h reoxygenation, whereas BV2 cells were treated with LPS for 24 h. Thereafter, cells were washed once with PBS, collected and the pulldown experiment was performed. RNA was extracted from the magnetic Dynabeads MyOne Streptavidin C1 (ThermoFisher Scientific, Waltham, USA) using mirVana miRNA Isolation Kit. RNA was reverse transcribed and cDNA templates were used for qPCR reactions with TaqMan gene expression assays (ThermoFisher Scientific, Waltham, USA). The data were normalized to control lysate values and then to fold changes calculated against control miRNA.

### Statistical analyses

Animals were randomized to treatment groups and procedures using GraphPad QuickCalcs online tool (GraphPad Software, San Diego, CA, USA). Data collected from the animal study were analyzed blinded to the treatment groups and the statistical analysis was run with GraphPad Prism 5.03 (GraphPad Software, San Diego, CA, USA) using either paired or unpaired two-tailed *t* tests or one-way ANOVA followed by Bonferroni post hoc tests to compare means of interest assuming homoscedasticity and normality of variables. Statistically significant outliers as calculated Grubb’s tests using the GraphPad Prism software were excluded from the datasets. Predetermined exclusion criteria for animals were bleeding during the surgeries, unsuccessful ischemia induction, or hemorrhages shown during MRI. Based on the exclusion criteria, none of the animals were excluded from pMCAo and tMCAo study. In total, eight animals from tMCAo study died: one from LV1-GFP and four from LV1-miR-669c group died during the ischemia surgery, two from LV1-GFP group died at 1 dpi and one at 2 dpi. No animals died from the pMCAo study. Cell culture experiments were repeated three times, and data was analyzed with unpaired two-tailed *t* test or one-way ANOVA followed by Bonferroni post hoc test. Data is reported as mean ± SEM unless otherwise stated and *N* numbers are stated in each figure legend. *P* values < 0.05 were considered statistically significant.

## Results

### MiR-669c-3p expression is increased upon excitotoxic or ischemic neuronal injury

**Fig. 1:**
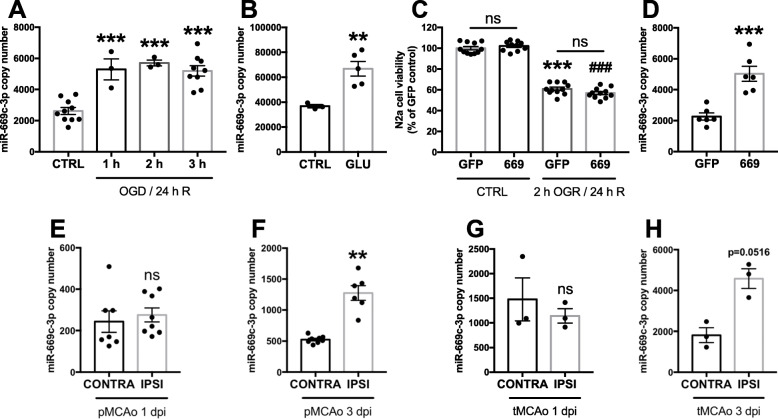
MiR-669c-3p expression is increased under conditions of OGD/R and ischemia. Quantitative real-time PCR for miR-669c-3p in N2a cells exposed to oxygen and glucose deprivation (OGD) for either 1, 2, or 3 h followed by reoxygenation for additional 24 h. **a** One-way ANOVA followed by Bonferroni’s post hoc tests, ***p < 0.001 compared to normoxic control cells (CTRL), *N* = 3-10. MiR-669c-3p expression is increased in primary cortical neurons exposed to excitotoxic injury (**b**). Quantitative real-time PCR for miR-669c-3p in primary cortical neurons treated with 400 μM glutamate (GLU) for 24 h. Unpaired two-tailed *t* test: **p < 0.01 compared to vehicle-treated primary cortical neurons (CTRL), *N* = 3-5. MiR-669c overexpression does not prevent the OGD/R-induced neuronal death in N2a cells (**c**). MTT assay of N2a cells exposed to OGD for 2 h followed by reoxygenation for 24 h. OGD/R reduced cell viability by approximately 40% as compared to normoxic cells. The assay was repeated three times with similar results. One-way ANOVA followed by Bonferroni’s post hoc tests: ***p < 0.001 or ^###^*p* < 0.001 compared to respectively LV1-GFP (GFP) and LV1-miR-669c (669) transduced normoxic cells (CTRL), *N* = 10-11. MiR-669c-3p expression is increased in LV1-miR-669c transduced N2a cells (**d**). Quantitative real-time PCR for miR-669c-3p in N2a cells transduced either with control LV1-GFP (GFP) or LV1-miR-669c (669). Unpaired two-tailed *t* test: ***p < 0.001 compared to LV1-GFP transduced cells, *N* = 6 in each group. Quantitative real-time PCR for miR-669c-3p in contralateral cortex (CONTRA) and peri-ischemic cortex area of the ipsilateral hemisphere (IPSI) at 1 (**e**) and 3 dpi (**f**) after pMCAo. Paired two-tailed *t* tests: **p < 0.01 compared to contralateral hemisphere, *N* = 6-8. Quantitative real-time PCR for miR-669c-3p in contralateral hemisphere (CONTRA) and ipsilateral hemisphere (IPSI) at 1 (**g**) and 3 dpi (**h**) after tMCAo. Paired two-tailed *t* tests, *N* = 3

To investigate whether OGD/R, glutamate exposure in vitro or ischemic stroke in vivo modulate the expression levels of miR-669c-3p, N2a cells were subjected to OGD for 1, 2, or 3 h followed by the reoxygenation for 24 h. OGD/R induced a significant increase of miR-669c-3p expression levels at all tested time points (Fig. 1a, *p* ≤ 0.001). Similarly, excitotoxic insult caused by 400 μM glutamate led to significant induction in miR-669c-3p expression in the primary cortical neurons (Fig. 1b, *p* = 0.009). To evaluate whether miR-669c-3p overexpression is directly neuroprotective in vitro, N2a cells were transduced with a lentiviral vector to overexpress miR-669c and exposed to OGD for 2 h followed by 24 h of reoxygenation. Overexpression of miR-669c failed to prevent ODG/R-induced N2a cell death (Fig. 1c). N2a cell transduction with LV1-miR-669c resulted in 2.21-fold upregulation in the expression of miR-669c-3p, compared to cells transduced with LV1-GFP control vector (Fig. 1d, *p* = 0.0004). To evaluate the extent of miR-669c expression in conditions of ischemic stroke in vivo, the levels of miR-669c were measured in two different models of stroke, pMCAo and tMCAo. pMCAo did not alter miR-669c-3p expression in the peri-ischemic (PI) area at 1 dpi (Fig. 1e) but the levels were elevated at 3 dpi (Fig. 1f, *p* = 0.0018), in comparison to the intact contralateral cortex. In tMCAo, the levels of miR-669c-3p remained unchanged at 1 dpi (Fig. 1g) but were nearly significantly increased at 3 dpi (Fig. 1h, *p* = 0.0516) in the ipsilateral hemisphere, as compared to the contralateral hemisphere. In contrast, the expression levels of the other arm of this miRNA hairpin precursor, miR-669c-5p, remained unchanged (*p* = 0.8385) between PI and contralateral cortex at 3 dpi in pMCAo model (data not shown).

### MiR-669c overexpression modulates the inflammatory response in BV2 cells by inducing anti-inflammatory microglial phenotype

**Fig. 2:**
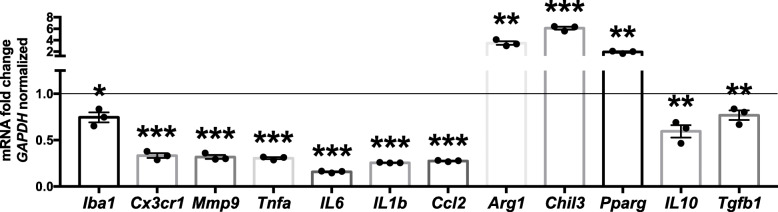
MiR-669c overexpression modulates BV2 microglial phenotype under LPS-induced inflammation. The expression of Iba1, CX3CR1, as well pro- and anti-inflammatory mediators MMP9, TNF-α, IL-6, IL-1β, CCL2, Arg1, Chil3/Ym1, PPAR-γ, IL-10, and TGF-β were analyzed by quantitative real-time PCR in LV1-GFP (control) and LV1-miR-669c transduced cells exposed to LPS (50 ng/ml) for 24 h. The assay was repeated three times with similar results. Values for miR-669c overexpressing cells normalized to LV1-GFP transduced (control) cells, presented as a solid line on graph. Unpaired two-tailed *t* tests: *p < 0.05, **p < 0.01, ***p < 0.001 compared to LV1-GFP transduced BV2 cells. *N* = 3 in each group

To assess the impact of miR-669c on microglial functions, we first measured the levels of miR-669c-3p in LPS and IL-4 exposed BV2 cells and LPS and IFN-γ/LPS exposed (referred as M1) primary murine microglia. Whereas LPS and IL-4 failed to alter the expression levels of miR-669c-3p (Supplementary Fig. 1A), IFN-γ in combination with LPS significantly induced the expression of miR-669c-3p in primary microglia (Supplementary Fig. 1B, *p* = 0.0003). To investigate whether astrocytes express miR-669c-3p to a similar extent as microglia, we assessed the levels of miR-669c-3p in both primary microglia and primary astrocytes. Astrocytes expressed significantly lower copy numbers of miR-669c-3p compared to primary microglia (Supplementary Fig. 1C, *p* = 0.0032). BV2 cell transduction with LV1-miR-669c resulted in 2.3-fold upregulation in the expression of miR-669c-3p compared to cells transduced with the control vector (Supplementary Fig. 1D, *p* = 0.0001). To evaluate whether miR-669c impacts the inflammation-related gene expression and cytokine release, BV2 cells were transduced with lentiviral vector to overexpress miR-699c and thereafter challenged with LPS (Fig. 2). Overexpression of miR-669c under proinflammatory conditions decreased the BV2 cell expression of Iba1 (*p* = 0.011), fractalkine receptor CX3CR1 (*p* = 0.0002), as well as proinflammatory genes MMP9 (*p* = 0.001), TNF-α (*p* < 0.001), IL-6 (*p* < 0.001), IL-1β (*p* < 0.001), and CCL2 (*p* < 0.001) compared to LV1-GFP transduced controls. Interestingly, it also induced a significant increase in the expression levels of microglia/macrophage alternative activation markers Arg1 (*p* = 0.0014), Chil3/Ym1 (*p* < 0.001), and PPAR-γ (*p* = 0.0071). On the contrary, the levels of immunosuppressive cytokines IL-10 (*p* = 0.0059) and TGF-β (*p* = 0.0143) were decreased in LV1-miR-669c transduced BV2 cells.

**Fig. 3:**
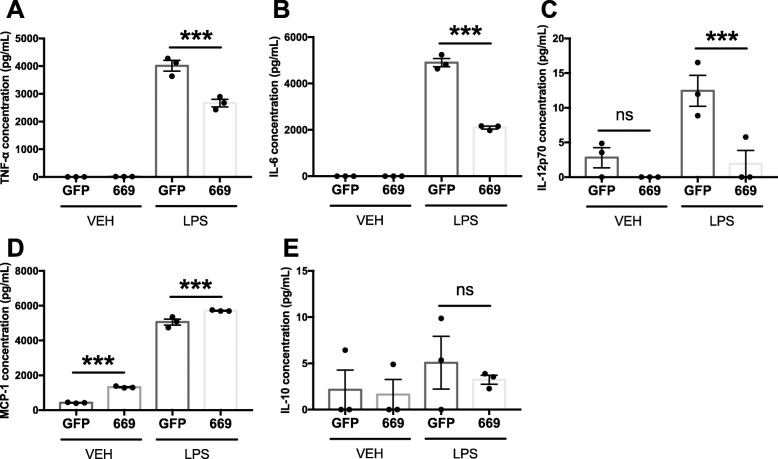
Inflammation-induced cytokine release is altered in miR-669c overexpressing BV2 cells. Proinflammatory cytokines IL-6 (**a**), MCP-1 (**c**), TNF-α (**d**), and IL-12p70 (**e**) and anti-inflammatory cytokine IL-10 (**b**) production was measured by CBA in the conditioned media from control LV1-GFP (GFP) or LV1-miR-669c (669) transduced cells exposed to vehicle or LPS (50 ng/ml) for 24 h. One-way ANOVA followed by Bonferroni’s post hoc tests: ***p < 0.001 compared to LV1-GFP transduced BV2 cells exposed to LPS. *N* = 3 in each group

To assess the impact of miR-669c on microglial cytokine production, LV1-miR-669c transduced BV2 cells were exposed to LPS and the secreted cytokines measured in the conditioned media. MiR-669c overexpressing cells demonstrated a decreased proinflammatory response to LPS exposure as the levels of TNF-α (Fig. 3a, *p* < 0.001), IL-6 (Fig. 3b, *p* < 0.001), and IL-12p70 (Fig. 3c, *p* = 0.0019) were significantly lower in miR-669c overexpressing BV2 cells compared to LPS-exposed LV1-GFP expressing controls. On the contrary, the levels of MCP-1 were increased (Fig. 3d, *p* = 0.0009) and levels of IL-10 (Fig. 3e) remained unaltered.

### MiR-669c overexpression in vivo decreased the ischemia-induced brain injury and ameliorated the neurobehavioral outcome

**Fig. 4:**
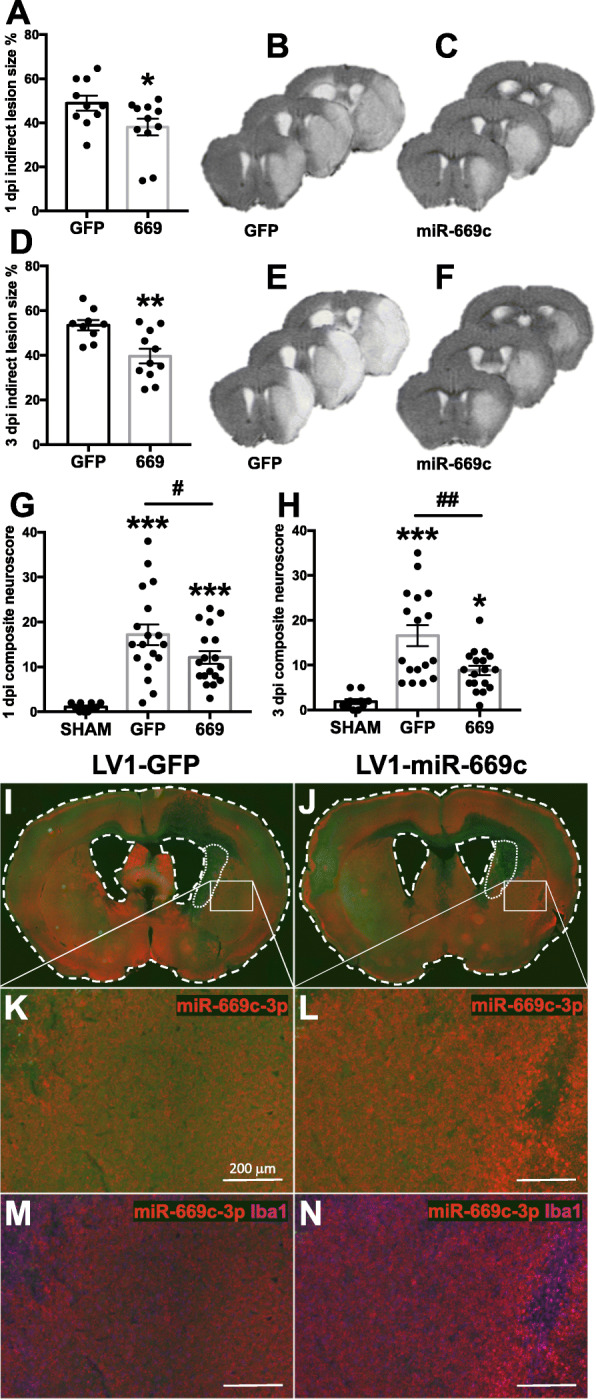
Lentiviral vector-mediated overexpression of miR-669c decreases ischemic brain damage and ameliorates neurological deficits. Quantification (**a**) and representative T2-weighted MRI brain images of tMCAo mice injected either with control LV1-GFP (**b**) or LV1-miR-669c (**c**) at 1 dpi. Respectively, panels **d**-**f** depict the quantification (**d**) and representative MRI images of control LV1-GFP (**e**) or LV1-miR-669c (**f**) animals 3 dpi. Unpaired two-tailed *t* tests: *p < 0.05, **p < 0.01 compared to LV1-GFP tMCAo mice. *N* = 9-11 in each tMCAo group. TMCAo animals intrastriatally injected with LV1-miR-669c (669) showed improved locomotor functions at 1 (**g**) and 3 dpi (**h**). Behavior was evaluated by composite neuroscore testing. One-way ANOVA followed by Bonferroni’s post hoc tests: *p < 0.05, ***p < 0.001 compared to sham-operated animals and ^#^p < 0.05, ^##^p < 0.01 compared to LV1-GFP tMCAo mice. *N* = 11 in sham-operated groups and *N* = 17-18 for tMCAo groups. Panels **i**-**n** illustrate the extent of miR-669c-3p expression (red) and Iba1 immunoreactivity (magenta) in representative LV1-GFP (**i**, **k**, **m**) or LV1-miR-669c (**j**, **l**,**n**) injected sham animals at 3 dpi. The LV injection sites are marked with white dotted lines

Finally, we investigated whether lentivirus-driven overexpression of miR-669c is neuroprotective in vivo against ischemia-induced cell death. LV1-miR-669c lentiviral vector was injected into the caudate putamen of C57BL/6 J mice. The animals were subjected to tMCAo 3 weeks post injection and the lesion volumes evaluated by MRI. The LV1-miR-669c injected mice showed significantly smaller ischemic damage both at 1 (*p* = 0.0489) and 3 dpi (*p* = 0.0044) (Fig. 4a-f). Concomitantly, the LV1-miR-669c injected mice showed markedly improved sensorimotor functions as analyzed by neuroscore testing at both time points (Fig. 4g, *p* = 0.0427 and 4H, *p* = 0.0014). Injection of LV1-miR-669c into the striatum increased the expression of miR-669c-3p within this region, when compared to control, LV1-GFP injected mice (Fig. 4i-n).

### LV1-miR-669c-mediated overexpression of miR-669c induces alternative microglial/macrophage activation and alters Iba1^+^ cell morphology in the ischemic brain

**Fig. 5:**
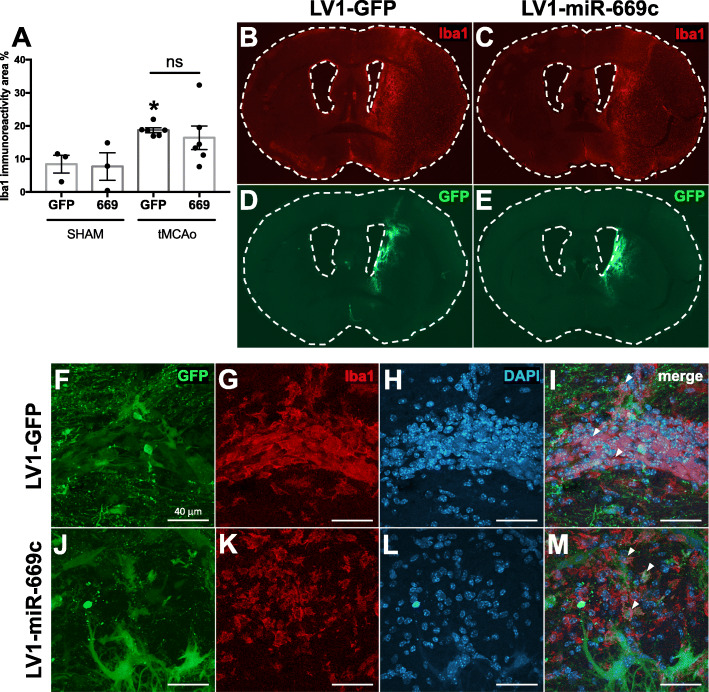
MiR-669c overexpression does not change overall microglia/macrophage activation under cerebral ischemia. Total microglial/macrophage activation was assessed by quantification of Iba1 immunostaining in the ipsilateral hemisphere. Iba1 immunoreactivity was not altered by the miR-669c-3p-mediated (669) overexpression at 3 dpi (**a**). Panels **b** and **c** are representative photographs of coronal sections stained with Iba1 in LV1-GFP control (**b**) and LV1-miR-669c injected tMCAo animals (**c**). One-way ANOVA followed by Bonferroni’s post hoc tests: *p < 0.05 compared to the respective sham-operated animals. *N* = 3 in each sham and *N* = 6 in each tMCAo group. Panels **d** and **e** depict the extent of GFP expression in the brains of the LV1-GFP (**d**) and LV1-miR-669c (**e**) injected stroke animals. Panels **f**-**m** contain confocal microphotographs depicting LV transduced, GFP^+^ cells colocalizing with Iba1^+^ cells in the ipsilateral striatum of LV1-GFP control (**f**-**i**) and LV1-miR-669c (**j**-**m**) stroke mice

**Fig. 6:**
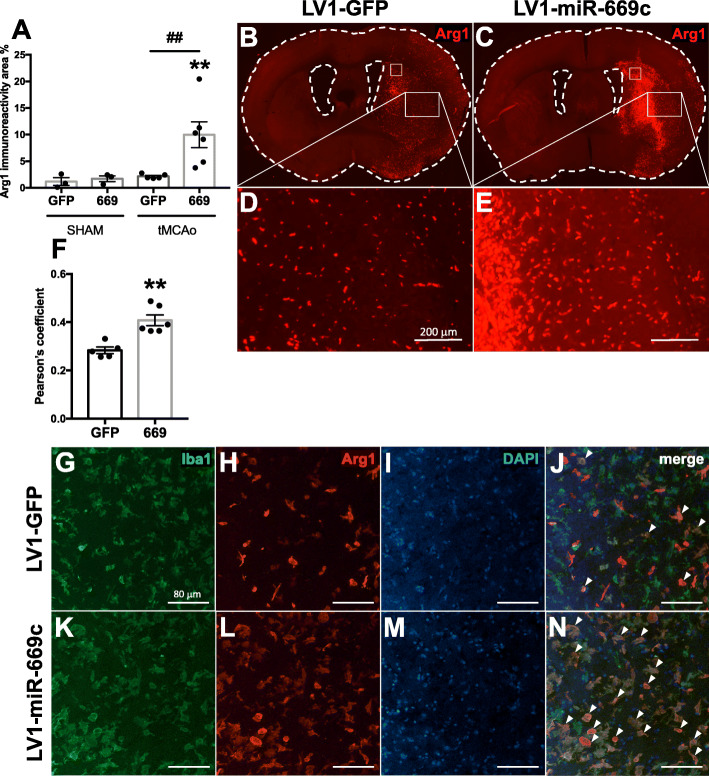
MiR-669c overexpression promotes microglial/macrophage alternative activation in ischemic brain. MiR-669c stroke group (669) showed significantly elevated levels of Arg1 immunoreactivity in the ischemic hemisphere compared to the LV1-GFP injected controls (GFP) at 3 dpi (**a**). Panels **b-e** show typical examples of Arg1 immunoreactivity in LV1-GFP control (**b**, **d**) or LV1-miR-669c (**c**, **e**) injected animals. The orange squares within panels **b** and **c** indicate the area imaged for Iba1/Arg1 colocalization analysis. One-way ANOVA followed by Bonferroni’s post hoc tests: *p < 0.05, **p < 0.01 compared to the respective sham-operated animals and ^##^*p* < 0.01 compared to control LV1-GFP tMCAo group. *N* = 3 in each sham and *N* = 6 in each tMCAo group. MiR-669c overexpression increases the colocalization of Arg1 and Iba1 in ischemic hemisphere (**f**). Panels **g**-**n** comprise confocal microphotographs representing Iba1^+^ and Arg1^+^ cells in the ipsilateral striatum of LV1-GFP control (**g**-**j**) and LV1-miR-669c (**k**-**n**) stroke animals. Unpaired two-tailed *t* test: **p < 0.01 compared to control LV1-GFP stroke animals. *N* = 5 for control and *N* = 6 for LV1-miR-669c stroke groups

To demonstrate the impact of LV1-miR-669c in ischemia-induced microglial activation, the brains of tMCAo animals were evaluated by IHC staining against typical microglial/macrophage marker Iba1 [38] and the levels of alternative activation marker Arg1 [39]. In LV1-GFP control group, stroke induced a significant upregulation in total Iba1 immunoreactivity (Fig. 5a, *p* = 0.0355). However, no significant change in the total Iba1 expression between the ischemic LV-miR-669c and LV1-GFP injected animals was observed in the ipsilateral hemisphere at 3 dpi (Fig. 5a-c, *p* = 0.5348). As expected, the extent of GFP signal around the LV injection site was comparable between LV1-GFP and LV1-miR-669c injected animals (Fig. 5d, e). The LV transduction led to GFP overexpression colocalizing with Iba1^+^ cells in both of the animal groups (Fig. 5f-m). Moreover, the LV-miR-669c injected stroke group had notably increased expression of Arg1 in comparison to LV1-miR-669c injected shams (Fig. 6a, *p* = 0.0083). Importantly, LV1-miR-669c injected ischemic mice demonstrated a significant upregulation in Arg1 immunoreactivity at 3 dpi compared to the control, LV1-GFP injected ischemic animals (Fig. 6a-e, *p* = 0.0044). Furthermore, miR-669c overexpressing ischemic animals showed increased cellular colocalization of Arg1 and Iba1, as indicated by Pearson’s correlation coefficient (Fig. 6f-n, *p* = 0.0016) and Mander’s overlap coefficient M2 (*p* = 0.0409, data not shown). The extent of overall CD45 immunoreactivity was unaltered in the ipsilateral hemispheres between the groups (Supplementary Fig. 2a-e, *p* = 0.1104) and double staining for Arg1 and CD45 revealed only a trend toward higher ratio of Arg1^+^ to round in shape, CD45^+^ cells in miR-669c overexpressing animals compared to LV1-GFP injected controls (Supplementary Fig. 2F-N, *p* = 0.0774). The CBA analysis of the cytokine concentration in plasma and brain homogenate samples failed to reveal significant alterations in the levels of proinflammatory cytokines between the groups (data not shown).

**Fig. 7:**
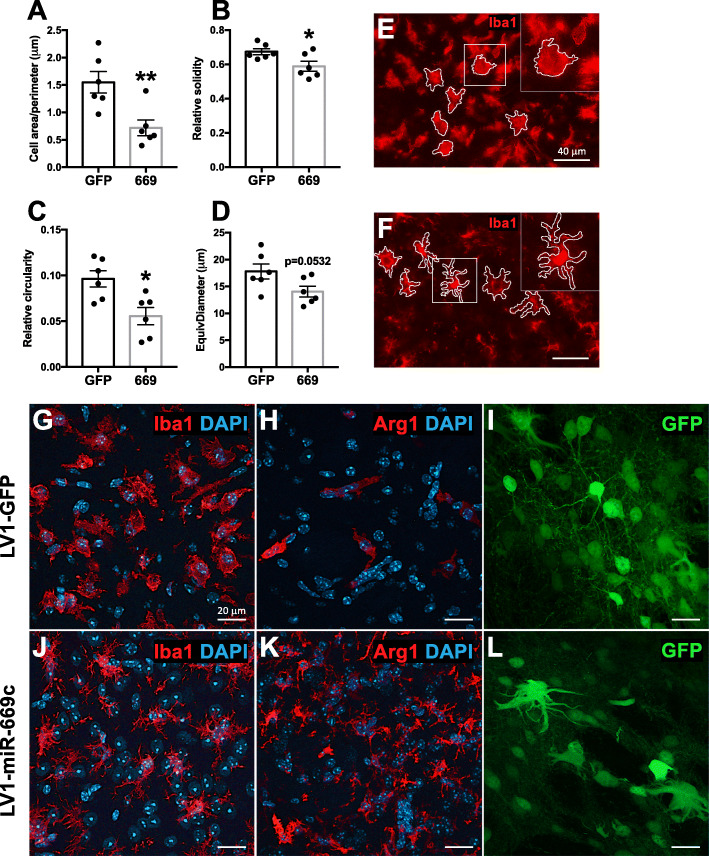
MiR-669c overexpression alters microglial morphology under ischemic stroke. Results of the cell morphology characteristic analysis in LV1-GFP (GFP) and LV1-miR-669c (669) animals: area/perimeter ratio (**a**), solidity (**b**), circularity (**c**), and EquivDiameter (**d**). The representative microphotographs of the infarct area depict the Iba1^+^ cell morphology in LV1-GFP (**e**), and LV1-miR-669c (**f**) overexpressing animals. Unpaired two-tailed *t* tests: *p < 0.05, **p < 0.01, compared to control group. *N* = 6 animals per each group. The representative confocal microphotographs taken from ipsilateral striatum illustrate the typical microglial morphology in Iba1 and Arg1 immunostained stroke brain sections of LV1-GFP (**g**, **h**) and LV1-miR-669c (**j**, **k**) overexpressing mice. Panels **i** and **l** represent the GFP overexpressing cells within the striatal area of LV1-GFP (**i**) and LV1-miR-669c (**l**) injected stroke animal

Although miR-669c overexpression did not change the total levels of Iba1 immunoreactivity in the brains of the ischemic mice, it significantly altered several morphological characteristics of the Iba1^+^ cells within the infarct site. The cell area/perimeter ratio (Fig. 7a, *p* = 0.0066), cell solidity (Fig. 7b, *p* = 0.0311), and circularity (Fig. 7c, *p* = 0.0107) were significantly lower, while the decrease of EquivDiameter failed to reach statistical significance (Fig. 7d, *p* = 0.0532) in LV1-miR-669c-injected animals as compared to the control group. Microglia in the resting state are typically characterized by long processes and relatively small soma. Upon activation, the microglial somas become larger and their processes shortened, leading to a decreased cell perimeter and thereby increased area/perimeter ratio [40]. Cell solidity is calculated as the ratio of cell area/convex area, whereas circularity value equal to 1 represents a perfect circle. Morphologically high circularity and solidity values correspond to cells with small number of membrane protrusions. In principle, the decrease in both of these factors denotes that Iba1^+^ cells from miR-669c group have smaller soma size, longer, and more prominent processes (Fig. 7f) in comparison to control animals (Fig. 7e). Confocal microphotographs depict differential morphology of intrastriatal Iba1^+^ and Arg1^+^ cells from control (Fig. 7g, h) and miR-669c overexpressing animals (Fig. 7j, k). Of note, based on the observed morphological traits, intracerebral injection of lentiviral vectors resulted in transduction of multiple cell types in the brain, including neurons, astrocytes, and microglia/macrophages (Fig. 7i, l).

### MiR-669c-3p targets the MyD88 transcript in vitro in BV2 microglial cells and in vivo levels of this target are decreased in miR-669c-3p overexpressing stroke mice

**Fig. 8:**
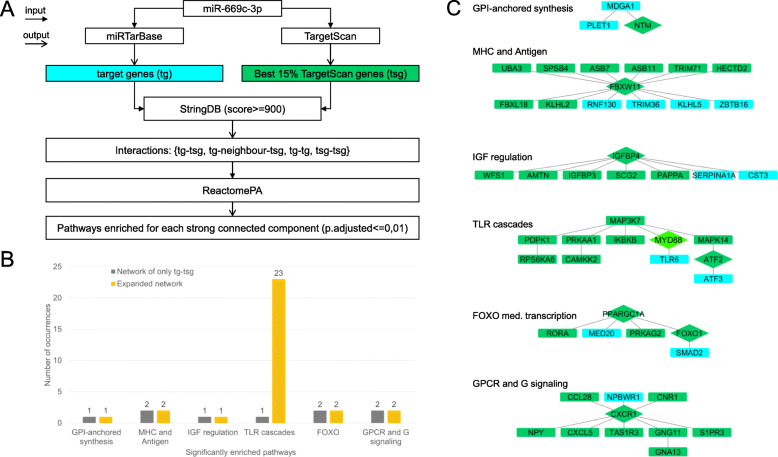
Neuroinflammation-related network analysis revealed MyD88 one of the most relevant predicted targets for miR-669c-3p. Panel **a** represents the workflow adapted in order to detect new genes significantly associated with miR-669c-3p. MiR-669c-3p was used as an input to query miRTarBase and TargetScan databases. Both experimentally validated targets from miRTarBase and TargetScan genes associated with miR-669c-3p were subsequently evaluated in STRING interaction database. Then a network was created, composed by the interactions between aforementioned targets depicted as nodes, as well as the interactions between TargetScan and miRTarBase targets including only those with high confidence (score ≥ 900). Finally, a pathway enrichment analysis of each connected component of the network was applied using the R package ReactomePA and only pathways enriched with an adjusted *p* value < 0.01 were retrieved. Panel **b** shows the results of pathway enrichment analysis prior (gray bars) and after (orange bars) the addition of interactions between TargetScan and miRTarBase targets in the network construction according to the workflow. When the network includes within-group interactions, only the connected component containing MyD88 (TargetScan target) and TLR6 (miRTarBase target) significantly increases the number of enriched neuroinflammatory pathways (23 Toll-like-receptor pathways). Panel **c** features the network obtained with adapted workflow. Diamond-shape green nodes are TargetScan targets directly connected to miRTarBase targets represented as rectangle-shape blue nodes. Rectangle-shape green nodes are the TargetScan targets connected to blue nodes due to the within-group interactions (green-green). Each subnetwork is one of the connected components enriched in pathways associated with neuroinflammation

**Fig. 9:**
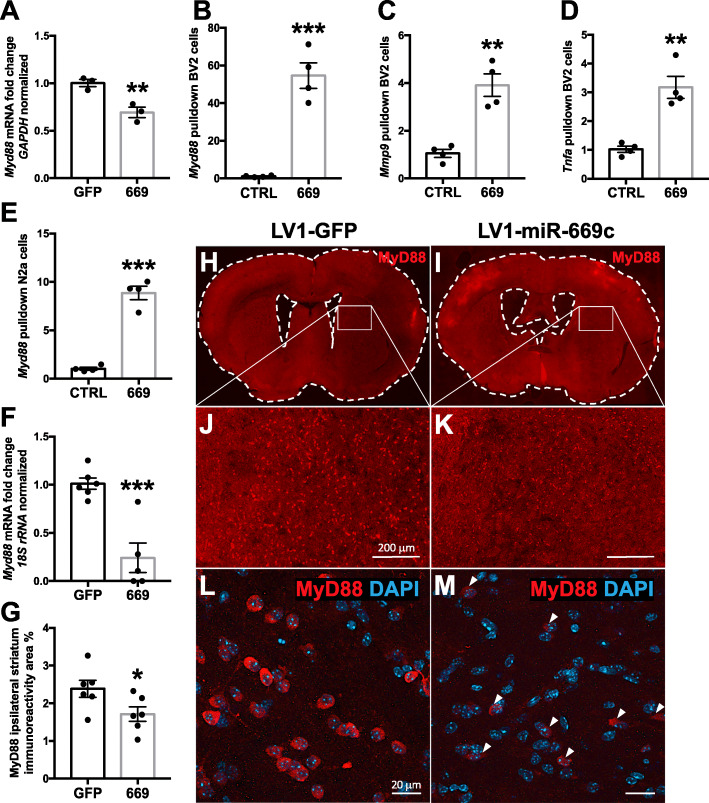
MiR-669c-3p is directly targeting MyD88 transcript in vitro and decreasing MyD88 immunoreactivity in ischemic stroke in vivo. The expression analysis of MyD88 has shown its downregulation in LPS-challenged miR-669c overexpressing BV2 cells (**a**). MiRNA pulldown results revealed that miR-669c-3p directly targets MyD88 (**b**), MMP9 (**c**), and TNF-α (**d**) transcripts in LPS exposed BV2 cells. MyD88 transcript also was shown to be targeted by miR-669c-3p in N2a cells exposed to OGD/R (**e**). The expression analysis of MyD88 confirmed its downregulation in miR-669c overexpressing N2a cells subjected to OGD/R (**f**). Unpaired two-tailed *t* tests: **p < 0.01, ***p < 0.001 compared to LV1-GFP transduced BV2 or N2a cells, ***p < 0.001 compared to control miR-39-3p transfected BV2 or N2a cells. *N* = 3-4 in each group. The extent of MyD88 ipsistriatal immunoreactivity was decreased in LV1-miR-669c injected stroke mice (**g**). Panels **h**-**m** depict typical examples of MyD88 immunoreactivity in LV1-GFP (**h**, **j**, **l**) and LV1-miR-669c (**i**, **k**, **m**) injected stroke animals. Unpaired two-tailed *t* test: *p < 0.05 compared to LV1-GFP stroke mice. *N* = 6 in each stroke group

In order to detect new significantly associated genes with miR-669c-3p, we took advantage of miRTarBase database and the TargetScan prediction tool. From miRTarBase, we manually retrieved experimentally validated miRNA-target interactions, whereas from TargetScan, we selected the best predicted targets of miR-669c-3p. Then, we performed a network analysis considering the aforementioned targets and their relationships in STRING with the highest score. Next, we carried out a pathway enrichment analysis of the connected components of the network in order to understand whether TargetScan genes were significantly enriched in pathways associated with neuroinflammation. The components containing genes Mdga1, Fbxw11, Igfbp4, Foxo1, Cxcr1, and MyD88 provided relevant neuroinflammation-associated pathways. Expanding the network with the relationships both among TargetScan targets and miRTarBase, the new pathway enrichment analysis highlighted only one component, containing i.a. MyD88 and Tlr6, strongly enriched in neuroinflammation-associated pathways and toll-like-receptor signaling (Fig. 8). Based on the results of network analysis, we selected MyD88 as the best predicted target and subsequently our pulldown analysis confirmed that MyD88 transcript is directly interacting with miR-669c-3p. Specifically, the results of pulldown assay revealed MyD88 as a direct target for miR-669c-3p both in BV2 (Fig. 9a, *p* = 0.0002) and in N2a cells (Fig. 9e, *p* < 0.001). We confirmed that MyD88 expression was decreased in both BV2 (Fig. 9b, *p* = 0.0101) and N2a cells (Fig. 9f, *p* = 0.0007) overexpressing miR-669c-3p. Other direct targets revealed using the pulldown assay for miR-669c-3p in BV2s were MMP9 (Fig. 9c, *p* = 0.0013) and TNF-α transcripts (Fig. 9d, *p* = 0.0016). The expression of other members of the toll-like receptor signaling pathway predicted as miR-669c-3p targets by the prediction tools, TLR4 (*p* = 0.314) and IRAK4 (*p* = 0.3442), were not changed and thus not targeted by miR-669c-3p (data not shown).

After identifying the MyD88 transcript as a direct target for miR-669c-3p in vitro, we finally validated its expression in the brains of ischemic mice. MyD88 immunoreactivity was significantly higher in the ipsilateral striatal area of control stroke group compared to sham-operated controls (data not shown, *p* = 0.029), in contrast to miR-669c-3p overexpressing stroke animals (data not shown, *p* = 0.7057). Although we failed to detect any significant changes of the total ipsilateral MyD88 levels (data not shown, *p* = 0.9918), ipsistriatal MyD88 immunoreactivity was specifically decreased in miR-669c-3p overexpressing animals (Fig. 9g-m, *p* = 0.0478).

## Discussion

Here, we show for the first time that overexpression of a hypoxia-inducible miR-669c mediates the neuroprotection and microglial/macrophage alternative activation in a mouse model of cerebral ischemia. Rather than altering the total Iba1 immunopositivity, miR-669c changed the morphology of Iba1 expressing cells and markedly increased the expression of Arg1, a marker for alternatively activated microglia and macrophages. A similar switch in microglial activation was also observed in vitro, where overexpression of miR-669c induced the expression of Arg1 and alleviated LPS-induced proinflammatory activation in BV2 cells. Furthermore, we discovered that miR-669c-3p directly interacts with MyD88, MMP9, and TNF-α transcripts.

Brain ischemia induces a rapid inflammatory response which is thought to be initiated and aggravated by the brain microglia, but also involves the infiltration of peripheral immune cells into the affected tissue. The complex intercellular crosstalk between the endogenous innate immune cells and the infiltrating leukocytes is initially meant to limit the ischemia-induced cell death, yet excessive proinflammatory activation has been shown to promote the neuronal apoptosis [41]. A vast amount of literature shows that modulation of inflammatory cascades is protective in cerebral stroke and these approaches are often mediated by an increase of microglial/macrophage alternative activation. The alternative activation phenotype is characterized by enhanced expression of the enzyme Arg1 [15, 42–45], which participates in endogenous tissue repair processes [46, 47] by supporting the extracellular matrix remodeling [48], axonal growth, and neuronal survival [49]. It has been shown that the majority of Arg1^+^ cells in the pMCAo model are infiltrating macrophages [50], which have also been suggested to be essential for maintaining the neuroprotective phenotype early after ischemic brain injury [51]. There have been studies demonstrating that in a tMCAo model miRNA-mediated Arg1 induction specifically in the microglia and macrophages provides the neuronal protection together with functional improvement [15]. In the current study, the neuroprotection in miR-669c overexpressing animals was also associated with a robust increase of Arg1 expression in the ischemic hemisphere at 3 dpi. These Arg1^+^ cells showed increased colocalization with Iba1 signal, indicating that they were primarily brain-resident immune cells, microglia, although we cannot exclude the contribution of infiltrating macrophages into the increased expression of Arg1 [52].

The ability of miRNAs to alter post-stroke gene transcription is well established. Sequencing studies have revealed that stroke induces alterations in levels of hundreds of miRNAs already during very early phases after the stroke onset. A number of studies have pinpointed miRNAs with the capacity to regulate stroke-induced neuroinflammatory responses for therapeutic benefit [53]. Inhibition or overexpression of miRNAs has been shown to limit the microglial activation and/or leukocyte infiltration, in many cases through targeting nuclear factor kappa B (NFκB) and thereby alleviating stroke-induced neuronal death. These include antagomirs for miR-22 [54] and miR-181a [14] and overexpression of miR-203 [55]. In addition, suppression of hypoxia-inducible miR-3473b [56] or upregulation of let-7c-5p [12] was shown to alleviate the microglial activation in vitro and in vivo, and to provide the protection in a mouse model of ischemic stroke. The fact that various miRNAs are capable of regulating the same pathways pinpoints the complexity of the regulation of post-stroke inflammation by miRNAs. Our data show that albeit miR-669c overexpression in N2a cells did not prevent the OGD/R-induced cell death in vitro, overexpression of miR-669c alleviated the microglial proinflammatory activation and increased the expression of alternative activation markers, indicating the modulation of inflammation toward a neuroprotective phenotype, both in vitro and in vivo. This was accompanied by a reduction in the lesion volume as well as amelioration of neurological deficits.

To our knowledge, this study is the first to describe that miR-669c-3p is induced upon ischemic stroke and to have a role in stroke-induced neuroinflammatory responses. In fact, miR-669c has been relatively little studied in the brain and only a handful of studies have pinpointed any role for this miRNA. Kuypers et al. described that the Sfmbt2 cluster is involved in the regulation of oligodendrocyte proliferation and remyelination [57]. In support of our data, the Sfmbt2 cluster miRNAs were shown to be upregulated in a rat model of tMCAo [22]. Interestingly, miR-669c-3p has been recently shown to interact with the network of circular RNAs in the transient ischemic stroke in mice [58]; however, the specific role of miR-669c-3p in cerebral ischemia has not been investigated further in detail. In addition, Druz et al. showed that the Sfmbt2 gene and miR-669c are induced following glucose deprivation-triggered oxidative stress and suggested that C2MC has a role in development of diseases involving oxidative stress [17, 18]. Similar to Druz et al., our data showed that miR-669c-3p is induced in both primary neurons and N2a cells exposed to glutamate or OGD/R, as well as now for the first time, in the cerebral ischemia in vivo*.* However, the overexpression of miR-669c did not further aggravate the OGD/R-induced neuronal death suggesting that this specific miRNA may have alternative functions in the conditions of ischemic stroke. Indeed, our study shows that instead of directly modulating the neuronal survival, miR-669c effectively regulates microglial inflammatory responses. Overexpression of miR-669c in microglial BV2 cells resulted in increased expression of alternative activation markers Arg1, Chil3 (Ym1), and PPAR-γ. Ym1, known also as chitinase-like 3, is considered a typical anti-inflammatory marker induced in macrophages and microglia in response to IL-4 treatment [59, 60]. It is interacting with heparin/heparan sulfate proteoglycans and mannose receptor, which are expressed by the alternatively activated microglia or macrophages, facilitating recovery and extracellular matrix remodeling [61]. Ligand-mediated peroxisome proliferator-activated receptor-γ activation has already been comprehensively described to promote microglia/macrophage alternative activation in various neuroinflammatory diseases [62–65]. Curiously, PPAR-γ was shown to directly bind to distal enhancer of Arg1 gene and thereby participate in regulation of alternative macrophage activation [66], and this mechanism could also be partially responsible for the protection seen in our model. Concomitantly, we observed a downregulation in several proinflammatory genes in miR-669c overexpressing, LPS-exposed BV2 cells, including Mmp9, Tnfa (TNF-α), Il6 (IL-6), Il1b (IL-1β), and Ccl2 (MCP-1). All of these factors have been extensively described in the literature as induced in the conditions of cerebral ischemia [67, 68]. To further investigate which neuroinflammation-related targets would be the most relevant for miR-669c-3p, we first carried out network analysis, which highlighted MyD88 as an important gene candidate for interaction with this miRNA, and then we confirmed with pulldown assay that miR-669c-3p indeed directly targets MyD88 transcript. Since the pulldown experiments revealed that in addition to MyD88, also MMP9 and TNF-α transcripts were direct targets of miR-669c-3p, it is very likely that the decrease in the expression of these inflammation-related factors specifically in microglia contribute to the protective effects observed in our study.

MyD88 acts as a key downstream signal transducing adaptor molecule in the TLR/NF-κB and IL-1/IL-1R1 signaling pathways [69], and recruits signaling proteins to the IFN-γ receptor, associated with the induction of innate immune response [70]. It is essential for the proper responses of IL-1, IL-18, and all TLRs, except TLR3, in the activation of transcription factors NF-κB and AP-1, followed by the induction of proinflammatory gene expression. It has been demonstrated that exaggerated or prolonged TLR-mediated inflammation can lead to aggravated pathology in various inflammatory diseases, such as sepsis, myocardial ischemia, and ischemic stroke [71–73]. Similarly, excessive stimulation of the innate immune system by MyD88 in certain pathologies may lead to overamplification of the inflammatory signaling [74]. It has been demonstrated that MyD88 signaling regulates leukocyte recruitment after brain injury and plays an important role in the regulation of early proinflammatory gene expression in this pathology [75]. Disruption of the TLR/MyD88/NF-κB signaling pathway was shown to be protective in the model of myocardial injury by attenuating NLRP3 inflammasome activation [76]. Moreover, alleviation or complete ablation of MyD88 signaling has been demonstrated as beneficial in several central nervous system pathologies, i.a., animal experimental autoimmune encephalomyelitis model [77], neuropathic pain [78], traumatic brain injury [79, 80], epilepsy [81, 82], Alzheimer's disease [83], hypoxic neonatal brain injury in LPS-sensitized mice [84], subarachnoid hemorrhage [85], as well as ischemic stroke [86, 87]. However, there is also a disparate evidence from another study indicating that the infarct size was not decreased in MyD88-/- mice subjected to permanent cerebral ischemia [88] and yet another study demonstrating that specifically hematopoietic cells exhibit a neuroprotective function after stroke and this is mediated by MyD88 [89]. Taken together, the functional outcome of modulation of TLR/MyD88 signaling pathway neuroinflammatory diseases requires careful fine-tuning and is likely to depend on the disease model, cell type, and additional regulation of MyD88-independent pathways. As it is difficult to develop inhibitors for adaptor proteins [90], miR-669c-3p-mediated inhibition of MyD88 may serve as a beneficial tool to control overactive TLR signaling in neuroinflammatory conditions.

The overexpression of miR-669c also normalized activated microglia and macrophage morphology. In healthy conditions microglial cells are characterized by highly ramified shape, enabling them to dynamically surveil the brain microenvironment [91]. Upon brain injury, these cells promptly respond to the pathological changes in the brain parenchyma and acquire ameboid shape, characterized by enlarged soma and retracted processes [92]. In comparison to the resting state, activated cells have a high cellular area to perimeter ratio, as they are rounded in shape and devoid of extensive branching. Iba1^+^ cells in vivo in miR-669c overexpressing ischemic animals showed a smaller area/perimeter ratio, as well as decreased solidity and circularity, and all of these parameters indicate that morphological changes of these cells could exhibit anti-inflammatory properties [30].

The expression of GFP driven by the lentiviral vectors was very local, yet the protection spanned throughout the ischemic hemisphere. This indicates that the miR-669c overexpression driven by the lentiviral construct may have spread throughout the ischemic brain. Accumulating amounts of literature suggest that this may occur via exosomes [93–95]. Although beyond the scope of this study, it is plausible that miR-669c spread within the ischemic brain via exosomal transfer, and thus the neuroprotection was observed throughout the ipsilateral hemisphere, and not only locally at the site of the lentiviral injection [96].

## Conclusions

Taken together, the current study is novel in multiple ways. We identify a novel hypoxia-regulated miRNA, miR-669c-3p, increased in conditions of cerebral stroke, and show that it is able to modulate microglial and macrophage activation toward anti-inflammatory phenotype. Importantly, miR-669c overexpression was able to reduce the ischemic brain damage and notably improve the neurobehavioral outcome of the stroke animals. We demonstrate novel binding partners for miR-669c-3p with the functional relevance in ischemic stroke and provide the evidence that this miRNA decreases in vivo expression of one of its targets, MyD88. However, it would be beneficial if less invasive treatment approaches than a direct intracerebral injection of miR-669c-overexpressing lentiviral vector could be tested in the near future, e.g., systemic delivery of miRNA mimics via RVG-exosomes or other lipid-based vehicles, which was proven successful for miR-124 [97]. Overall, our findings emphasize the importance and potential of miRNAs used as a tool for successful control of neuroinflammatory responses in the context of cerebral ischemia.

## Supplementary information

**Additional file 1: Supplementary Figure 1.** MiR-669c-3p levels are upregulated in primary murine microglia upon LPS and IFN-γ challenge. LPS or IL-4 exposure alone does not alter BV2 cell (A) or primary microglial cell (B) expression of miR-669c-3p, yet miR-669c-3p expression is induced in primary microglia upon IFN-γ and LPS combined stimulation (M1) (B). Quantitative real-time PCR for miR-669c-3p in wild type BV2 cells treated with vehicle, LPS (50 ng/ml) or IL-4 (20 ng/ml) for 24 h or primary murine microglia treated with vehicle or IFN-γ (20 ng/ml) in combination with LPS (10 ng/ml) for 24 h. One-way ANOVA followed by Bonferroni's post-hoc tests: N = 6-7 in each group. MiR-669c-3p expression is higher in unstimulated primary microglia in comparison to primary astrocytes (C). Quantitative real-time PCR for miR-669c-3p in vehicle-treated primary murine microglia (MG) or vehicle primary murine astrocytes (ASTRO). Unpaired two-tailed t-test: **p < 0.01 compared to vehicle treated astrocytes, N = 3-6. MiR-669c-3p (669) expression is increased in LV1-miR-669c transduced BV2 cells versus LV1-GFP (GFP) transduced cells (D). Quantitative real-time PCR for miR-669c-3p in BV2 cells transduced either with control LV1-GFP or LV1-miR-669c. Unpaired two-tailed t-test: ***p < 0.001 compared to LV1-GFP transduced vehicle, N = 4-6 in each group.

**Additional file 2: Supplementary Figure 2.** CD45 expression does not change under miR-669c overexpression in conditions of brain ischemia. The ipsilateral CD45 immunoreactivity remained unaltered between stroke control LV1-GFP (GFP) and LV1-miR-669c mice (669) (A). Panels B-E are representative photographs of coronal sections stained with CD45 in LV1-GFP control (B, D) and LV1-miR-669c injected tMCAo animals (C, E). Similarly, the ratio of Arg1^+^ to round in shape, bright CD45^+^ cells was not changed in LV1-miR-669c animals (669) comparing to the control group (GFP) (F). Panels G-N consists of confocal microphotographs illustrating the proportion of Arg1^+^ and CD45^+^ cells in the ipsilateral striatum of LV1-GFP control (G-J) and LV1-miR-669c (K-N) stroke animals. Unpaired two-tailed t-tests. N = 6 animals per each group.

## Data Availability

All data acquired during the study is available from the corresponding author upon reasonable request.

## Abbreviations

Arg1: Arginase 1
C2MC: Chromosome 2 miRNA cluster
CBA: Cytometric bead array
CCA: Common carotid artery
CCL2: Monocyte chemotactic protein 1 (MCP-1)
CD45: Leukocyte common antigen
CX3CR1: CX3C chemokine receptor 1
DAPI: 4′,6-diamidino-2-phenylindole
DIV: Day in vitro
dpi: Days post-injury
ECA: External carotid artery
EDC: 1-ethyl-3-[3-dimethylaminopropyl] carbodiimide hydrochloride
EDTA: Ethylenediaminetetraacetic acid
EGTA: Egtazic acid
FISH: Fluorescent in situ hybridization
GAPDH: Glyceraldehyde 3-phosphate dehydrogenase
GFP: Green fluorescent protein
Iba1: Ionized calcium-binding adapter molecule 1 (Aif1)
ICA: Internal carotid artery
iFBS: Heat inactivated fetal bovine serum
IFN-γ: Interferon gamma
IHC: Immunohistochemistry
IL: Interleukin
IP: Intraperitoneally
IRAK4: Interleukin-1 receptor-associated kinase 4
LPS: Lipopolysaccharide
LV: Lentiviral vector
MCA: Middle cerebral artery
MMP9: Matrix metalloproteinase 9
MOI: Multiplicity of infection
MRI: Magnetic resonance imaging
MyD88: Myeloid differentiation primary response gene 88
NFκB: Nuclear factor kappa B
OGD/R: Oxygen and glucose deprivation/reoxygenation
pMCAo: Permanent middle cerebral artery occlusion
PBS(T): Phosphate buffered saline (with Tween 20)
PFA: Paraformaldehyde
PPAR-γ: Peroxisome proliferator-activated receptor-γ
RVG: Rabies virus glycoprotein
Sfmbt2: Sex comb on the midleg with four MBT domains-2
TE: Tris-EDTA
TGF-β: Transforming growth factor beta
TLR: Toll-like receptor
TNF-α: Tumor necrosis factor alpha
tMCAo: Transient middle cerebral artery occlusion
TU: Transducing units
UTR: Untranslated region
Ym1: Chitinase-like 3 (Chil3)
