# Supplementary figures and images for: Intracerebral overexpression of miR-669c is protective in mouse ischemic stroke model by targeting MyD88 and inducing alternative microglial/macrophage activation
Source: J Neuroinflammation. 2020 Jun 19;17:194. doi: 10.1186/s12974-020-01870-w (PMC7304130; doi:10.1186/s12974-020-01870-w)

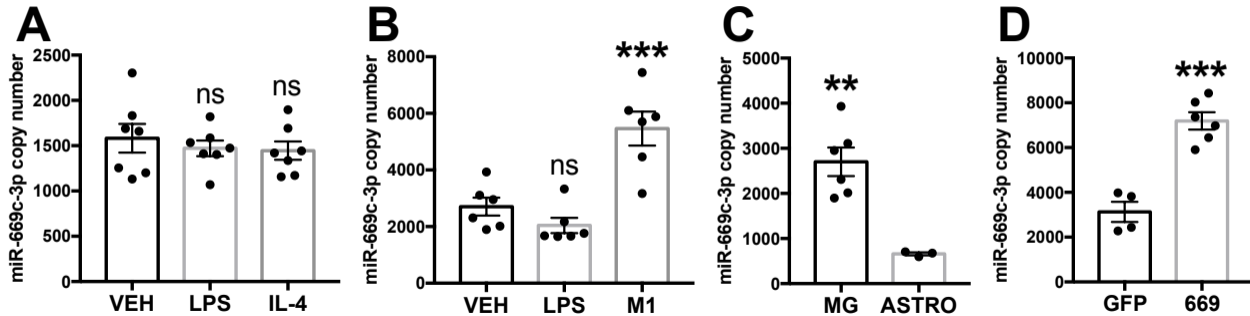

Supplement: Supplementary file 1 — Additional file 1: Supplementary Figure 1. MiR-669c-3p levels are upregulated in primary murine microglia upon LPS and IFN-γ challenge. LPS or IL-4 exposure alone does not alter BV2 cell (A) or primary microglial cell (B) expression of miR-669c-3p, yet miR-669c-3p expression is induced in primary microglia upon IFN-γ and LPS combined stimulation (M1) (B). Quantitative real-time PCR for miR-669c-3p in wild type BV2 cells treated with vehicle, LPS (50 ng/ml) or IL-4 (20 ng/ml) for 24 h or primary murine microglia treated with vehicle or IFN-γ (20 ng/ml) in combination with LPS (10 ng/ml) for 24 h. One-way ANOVA followed by Bonferroni's post-hoc tests: N = 6-7 in each group. MiR-669c-3p expression is higher in unstimulated primary microglia in comparison to primary astrocytes (C). Quantitative real-time PCR for miR-669c-3p in vehicle-treated primary murine microglia (MG) or vehicle primary murine astrocytes (ASTRO). Unpaired two-tailed t-test: **p < 0.01 compared to vehicle treated astrocytes, N = 3-6. MiR-669c-3p (669) expression is increased in LV1-miR-669c transduced BV2 cells versus LV1-GFP (GFP) transduced cells (D). Quantitative real-time PCR for miR-669c-3p in BV2 cells transduced either with control LV1-GFP or LV1-miR-669c. Unpaired two-tailed t-test: ***p < 0.001 compared to LV1-GFP transduced vehicle, N = 4-6 in each group. [file 12974_2020_1870_MOESM1_ESM.pdf]

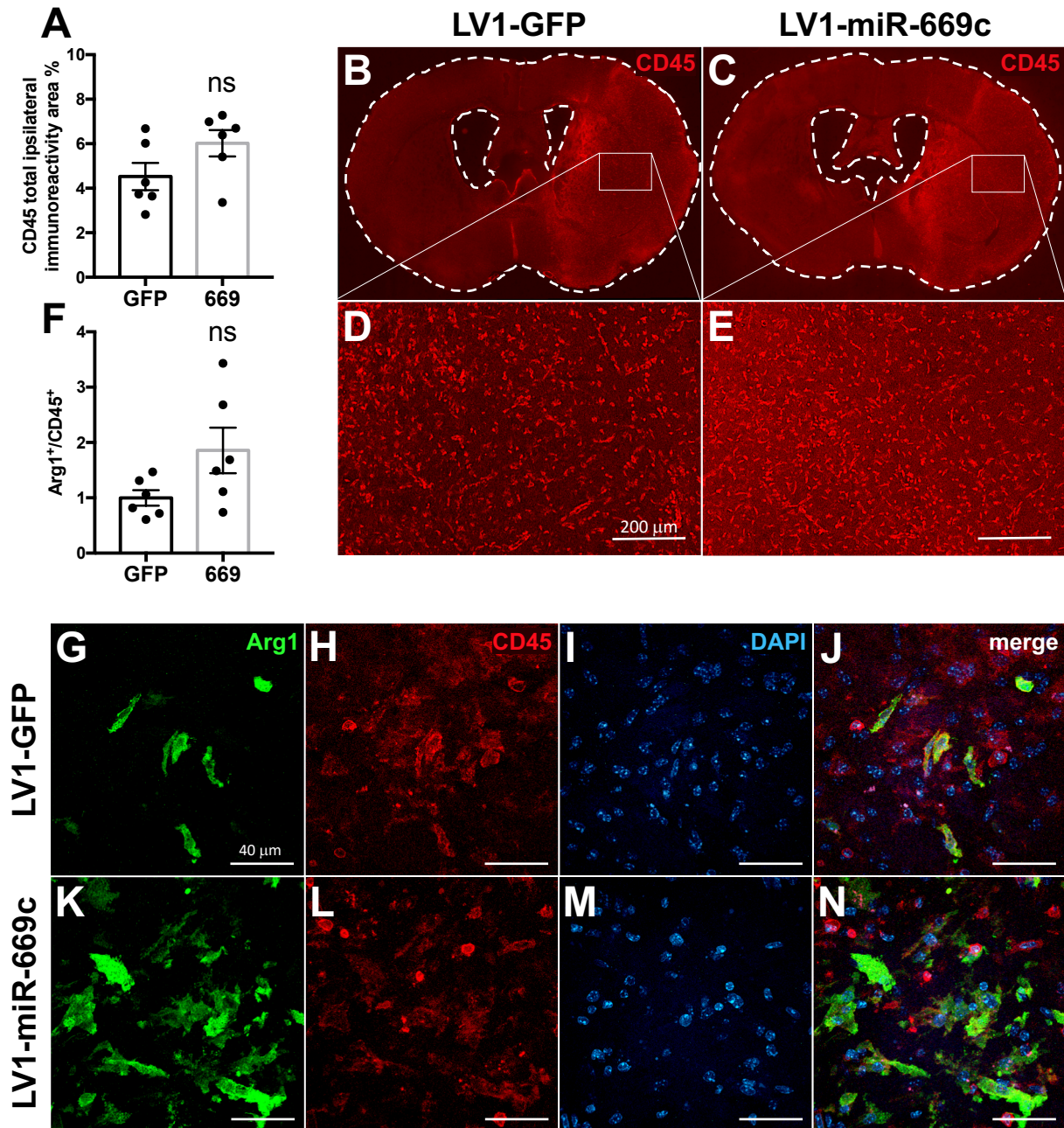

Supplement: Supplementary file 2 — Additional file 2: Supplementary Figure 2. CD45 expression does not change under miR-669c overexpression in conditions of brain ischemia. The ipsilateral CD45 immunoreactivity remained unaltered between stroke control LV1-GFP (GFP) and LV1-miR-669c mice (669) (A). Panels B-E are representative photographs of coronal sections stained with CD45 in LV1-GFP control (B, D) and LV1-miR-669c injected tMCAo animals (C, E). Similarly, the ratio of Arg1+ to round in shape, bright CD45+ cells was not changed in LV1-miR-669c animals (669) comparing to the control group (GFP) (F). Panels G-N consists of confocal microphotographs illustrating the proportion of Arg1+ and CD45+ cells in the ipsilateral striatum of LV1-GFP control (G-J) and LV1-miR-669c (K-N) stroke animals. Unpaired two-tailed t-tests. N = 6 animals per each group. [file 12974_2020_1870_MOESM2_ESM.pdf]
